# Is there structural change on MRI in gluteal tendinopathy after treatment? Single outcome measure extension of an RCT

**DOI:** 10.1186/s12880-023-01150-y

**Published:** 2023-11-08

**Authors:** Georgia Carney, Jane Fitzpatrick

**Affiliations:** 1 Faculty of Medicine, Dentistry and Health Sciences, The University of Melbourne, Level 7, Alan Gilbert Building, 161 Barry Street, Victoria, 3010 Australia; 2 Joint Health Institute, Richmond, Melbourne, Australia; 3 Australasian College of Sports and Exercise Physicians, Melbourne, Australia

**Keywords:** Platelet-rich plasma, Ortho-biologics, Regenerative medicine, Sports medicine

## Abstract

**Background:**

The etiology of tendinopathy remains controversial and it is unknown whether degenerative structural changes in tendinopathies are reversible.

**Hypothesis:**

There will be no structural change on magnetic resonance imaging (MRI) taken > 2-years after treatment for gluteal tendinopathy.

**Study Design:**

Extension of a single site, double-blind, prospective randomized-controlled trial to analyze the additional outcome measure; MRI changes.

**Methods:**

University of Melbourne ethics approval number: 1852900, trial registration: ACTRN12613000677707. Participants with gluteal tendinopathy who had previously received a leukocyte-rich platelet-rich plasma injection (LR-PRP) or a corticosteroid injection (CSI) had a post treatment MRI between at least 2-years and up to 7 years following trial completion. A blinded, senior musculoskeletal radiologist graded all de-identified MRI scans using the Melbourne Hip Score (MHIP). The primary outcome measure was the change in overall pre- and post-treatment score.

**Results:**

Participants (n = 20) underwent MRI at mean time of 4.15 (SD 1.11; range 2–7) years after their initial treatment. There was no change in the overall mean MHIP score for the CSI group (Pre 4.3 (SD 2.3) Post 4.3 (SD 1.1), p = 1.00). Although there was an improvement in the LR-PRP group mean MHIP score (Pre 5.3 (SD 3.0) Post 4.77 (SD 2.5), p = 0.56) it was not statistically significant. However, in the LR-PRP intervention group, five out of nine of participants’ MHIP score improved, with four of these improving by 2–4 points.

**Conclusion:**

The hypothesis that there would be no improvement in MHIP scores following treatment of gluteal tendinopathy was supported. Findings of improvement in the LR-PRP group at 4 years would support further studies powered to look for structural improvement. These findings suggest that structural change following treatment for tendinopathy may be possible supporting the inclusion of MRI as a core outcome for future studies.

**Clinical relevance:**

The study suggests that degenerative structural changes in tendons may be reversible.

**Supplementary Information:**

The online version contains supplementary material available at 10.1186/s12880-023-01150-y.

## Significance

*What is known about this subject?* Carbon dating techniques have determined that the collagen matrix within tendon architecture is a permanent structure laid down well before adulthood. Given the poor correlation between tendinopathy diagnoses and radiological findings, there is an assumption that structural changes seen radiologically are not related to tendinopathic symptoms. Thus, tendon research has focused on targeting symptomatic, rather than structural, improvement.

*What this study adds to existing knowledge?* Using the Melbourne Hip MRI (MHIP) scoring system we conducted a long-term follow-up with participants from a single site, double-blind, prospective randomized-controlled trial to analyze the additional outcome measure, structural MRI changes. Participants who had received a leukocyte-rich platelet-rich plasma (LR-PRP) or corticosteroid injection (CSI) treatment for gluteal tendinopathy underwent MRI at a mean time of 4.15 (SD 1.11; range 2–7) years post-treatment. More than half of the participants showed structural changes on their MRI.

**Supplementary Information:**

The online version contains supplementary material available at 10.1186/s12880-023-01150-y.

## Introduction

Accounting for up to 30% of all musculoskeletal consultations [1] tendinopathies are caused by overuse and repetitive movements such as during sports and exercise or occupational loading.

Whilst tendinopathy was originally considered an ‘inflammatory’ condition, the pathophysiology of musculoskeletal diseases is being re-considered.

Recent molecular evidence, has acknowledged the inflammatory nature of tendinopathy with key inflammatory processes occurring even before symptoms develop [2]. Tenocytes react to external stimuli such as loading and have been shown to synthesize new extracellular matrix under specific anabolic loading conditions [2]. Further, there is evidence that type III collagen is laid down in the early phase of tendon repair before being converted to type I collagen [3]. Heinemeier et al. showed that collagen undergoes virtually no turnover in healthy adult tendons but is replaced at an abnormally high rate in tendinopathic tissue [4]. Jarvienen described the associated process of neovascularization in tendinopathic tissue [5] and Bhabra et al. introduced a histopathological model of tendinopathy [6]. However, even where treatments such as exercise, have been shown to reduce symptoms, there have not been any changes in structural improvements to explain this symptomatic improvement [7].

A recent randomized controlled trial (RCT) study by Fitzpatrick et al. assessed the effectiveness of leukocyte-rich platelet-rich plasma (LR-PRP) injections in the management of gluteal tendinopathy [8, 9]. The trial showed that patients with a history of chronic gluteal tendinopathy of more than 14 months experienced significant improvement in their mean modified Harris Hip Scores (mHHS) over the first three months. The authors noted that the patients continued to improve symptomatically beyond 12 months and even further at final follow up at 24 months [8, 9]. No imaging or structural outcome measures were used at the time based on the prevailing theory that the degenerative changes in tendons were essentially irreversible.

Limited studies to date have investigated if, and to what extent, structural change occurs on MRI in tendinopathic tendons following treatment [10, 11]. The aim of this study was to measure structural change in pre- and post- treatment magnetic resonance imaging (MRI) scores using the Melbourne Hip MRI Score (MHIP) [12]. Our hypothesis was that there would be no improvement in radiological appearance consistent with the Bhabra et al. model of tendinopathy disease progression [6].

## Methods

### Ethics approval

The study was conducted in accordance with the Declaration of Helsinki and Ethics approval was obtained from the University of Melbourne Human Research Ethics Committee (Ethics approval number: 1852900). The trial was registered with ACTRN12613000677707. All participants provided written informed consent to participate.

### Study design

This study is an extension of a single site (Melbourne), double-blind, prospective RCT to analyze the structural outcome measure of MRI changes at 2-years or more post treatment in addition to the previously reported clinical outcomes measures [8, 9]. CONSORT guidelines (Consolidated Standards of Reporting Trials) were followed in the reporting of this trial [13].

### Participants

Participants were drawn from the existing RCT comparing LR-PRP to corticosteroid injection (CSI) for gluteal tendinopathy [8, 9]. Inclusion criteria were all participants who had available baseline magnetic resonance imaging (MRI). Exclusion criteria was inability to undertake an MRI.

### Intervention

No treatment was given to the participants in this study although participants had previously received a single LR-PRP injection (group 1), a single CSI (group 2) or a single CSI followed by a LR-PRP injection (group 3) as described by the methodology and protocol of Fitzpatrick et al. [9] Participants were asked to have a post treatment MRI at > 2-years following completion of the trial.

Where possible, scans were taken on the same machine. The MRI machine used a Skyra 3-T superconducting unit and Numaris/4 Syngo MR 11 software (Siemens), with a slew rate of 200 T/m/s, 45 mT/m gradient amplitude with a high-resolution 18-channel surface coil anteriorly and the 32-channel body coil posteriorly. Multiplanar sagittal, axial, and coronal proton density and fat saturated T2 weighted images were taken. Where it was not possible for participants to access this machine, scans were performed using standard protocols based on the European Society of Skeletal Radiology guidelines [14].

### Outcome measure

A blinded, senior musculoskeletal radiologist graded all de-identified MRI scans using the Melbourne Hip Score (MHIP). The severity of soft tissue bursitis and direct gluteal tendon pathology has been shown to affect the adjacent structures, notably fatty infiltration of muscle and bone marrow oedema. Thus, the MHIP score was designed as a holistic score to fully represent the severity of tendinopathy by including both the direct measures of tendon pathology and the indirect measures in the adjacent muscle and bone [11]. The MHIP score has a total of 17 points derived from 5 elements each of which in total, has been shown to reflect tendinopathy severity: the extent of gluteal tendinopathy (5 points); trochanteric bursitis (4 points); cortical irregularity (3 points); muscle fatty atrophy (4 points) and bone marrow oedema (1 point) [12]. The MHIP score has excellent intra-observer reliability for determining the severity of gluteal tendinopathy with an interclass correlation coefficient of 0.81 (95% CI, 0.67–0.89) [12]. This study was performed with the same radiologist and the same baseline films read in the MHIP reliability study, thus we can be sure of the intra-observer reliability and the need to have each MRI graded more than once. The primary outcome measure was the change in overall pre- and post-treatment MHIP scores. Since there is no previously published data on changes in MHIP scores, the authors determined a change in score of + 2 or more to reflect structural improvement. A change of less than 2 or a negative change in score (reflecting worsening) was reported as no structural improvement.

### Statistical analysis

Analysis was conducted using STATA version 13.1 (Stata Corp. 2016 Stata Statistical Software: Release 13.1. College station, TX: Stata Corp LP). Classical 2-sided paired student t-tests were performed to determine if there was a statistically significant difference in the change in pre-and post-treatment score within the trial groups. A sample size calculation performed using www.gigacalculator.com for difference between samples using continuous means and relative difference (Type I error rate of 5%, power of 80%, mean 5 (SD 2.5) and MDE 0.5) gives a sample size of 26 (13 in each group). As there were 76 participants in the RCT from which participants were drawn, the sample size was accepted.

## Results

### Flow of patients

**Fig. 1:**
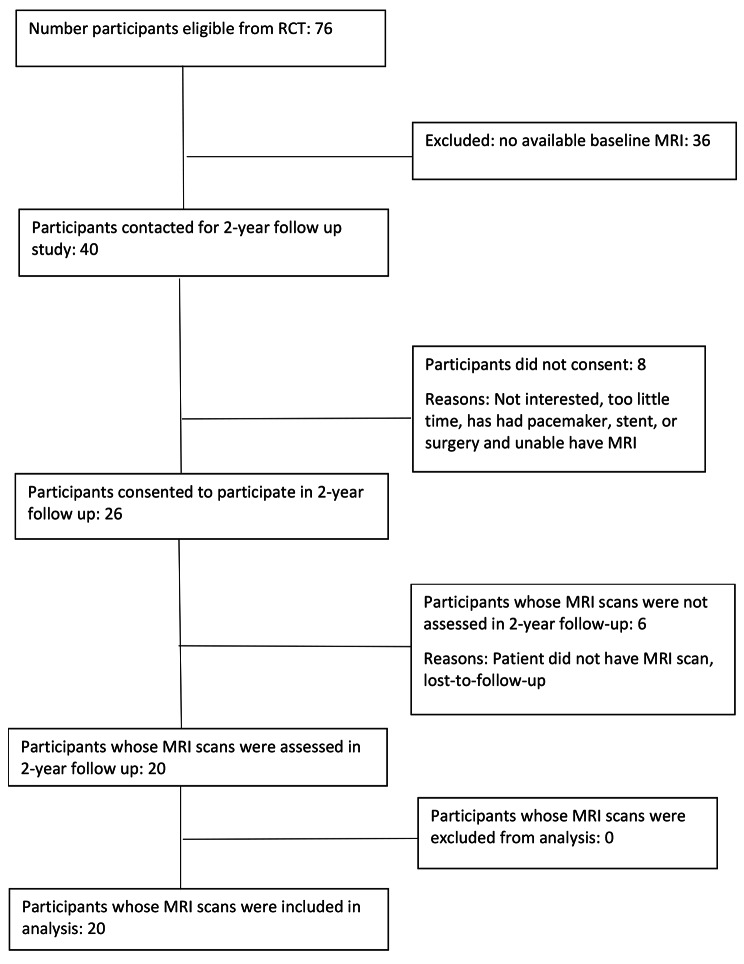
MHIP scores graphical representation. MHIP, Melbourne Hip MRI Score; LR-PRP, leukocyte-rich platelet-rich plasma; CSI, corticosteroid injection

Of 76 participants who were eligible for this study, 36 were excluded as they did not have available baseline MRI scans and 8 did not consent to participate (Fig. 1). 6 participants did not have their MRI (noting there were significant COVID-19 lockdowns affecting the regions in which participants lived) leaving 20 participants with 40 MRI scans available for analysis. There were 19 females and one male with a mean age of 58 years (SD 10, range 36–75 years) (Table 1).

**Table 1:** Demographic data

Participants, total 20	
Age, mean (range), y	58.45 ± 10.63 (36–75)
Sex, n (%)	
Male	1 (5.0)
Female	19 (95.0)
Body mass index, mean ± SD (range), kg/${m}^{2}$	28.46 ± 5.65 (20.2–43.9)
Baseline mHHS, mean ± SD (range)	53.35 ± 9.27 (37–74)

Y, years; N, number; SD, standard deviation; kg, kilograms, m, meters; mHHS, modified Harris Hip Score.

### Time to post-treatment MRI

Participants received post-treatment MRI at a mean time of 4.15 (SD 1.11; range 2–7) years after their initial treatment.

### Change in MHIP score overall

Table 2 shows the subgroup scores and the total for the cohort of changes in MHIP scores. Overall, the majority of patients (11/20) showed no structural improvement based on MHIP scores. Given 9 patients showed some improvement, the subgroup analysis was reported, despite the small sample size, to see if all groups were similar or there was any indication of treatment related change. The authors postulated that since CSI have been suggested to cause further degeneration in tendons (leading to rupture) [15], there may be a difference between the CSI group and other interventions.

There was no change in the overall mean MHIP score for the CSI group (Pre 4.3 (SD 2.3) Post 4.3 (SD 1.2)) but there was an improvement in the LR-PRP group mean MHIP score (Pre 5.3 (SD 3.0) Post 4.8 (SD 2.5), p = 0.56) (supplementary tables S1 and S2).

**Fig. 2:**
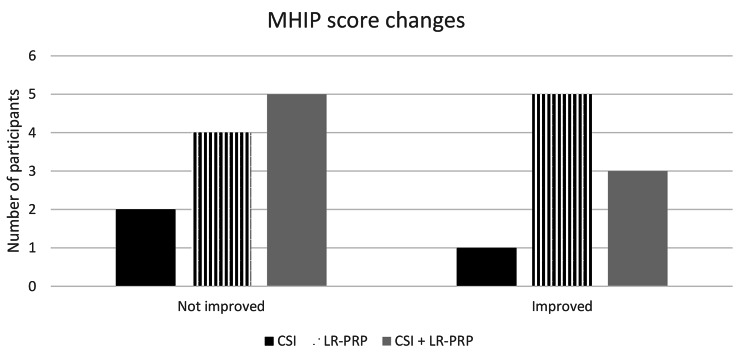
MHIP scores graphical representation. MHIP, Melbourne Hip MRI Score; LR-PRP, leukocyte-rich platelet-rich plasma; CSI, corticosteroid injection

Figure 2 shows the number of participants who either improved or did not in each group. In the CSI group most participants had no change or a worsened MHIP score over > 2 years. However, in the LR-PRP intervention group, 5/9 participants MHIP score improved, with four of these improving by between 2 and 4 points.

**Table 2:** MHIP scores by treatment group comparison

	No. of patients no structural improvement	No. of patients Structural improvement	Total no. of patients	% MHIP not structurally improved	% MHIP Structurally Improved
CSI	2	1	3	67	33
LR-PRP	4	5	9	44	56
CSI + LR-PRP	5	3	8	63	37
Total	11	9	20	55	45

MHIP, Melbourne Hip MRI Score; LR-PRP, leukocyte-rich platelet-rich plasma; CSI, corticosteroid injection.

**Fig. 3:**
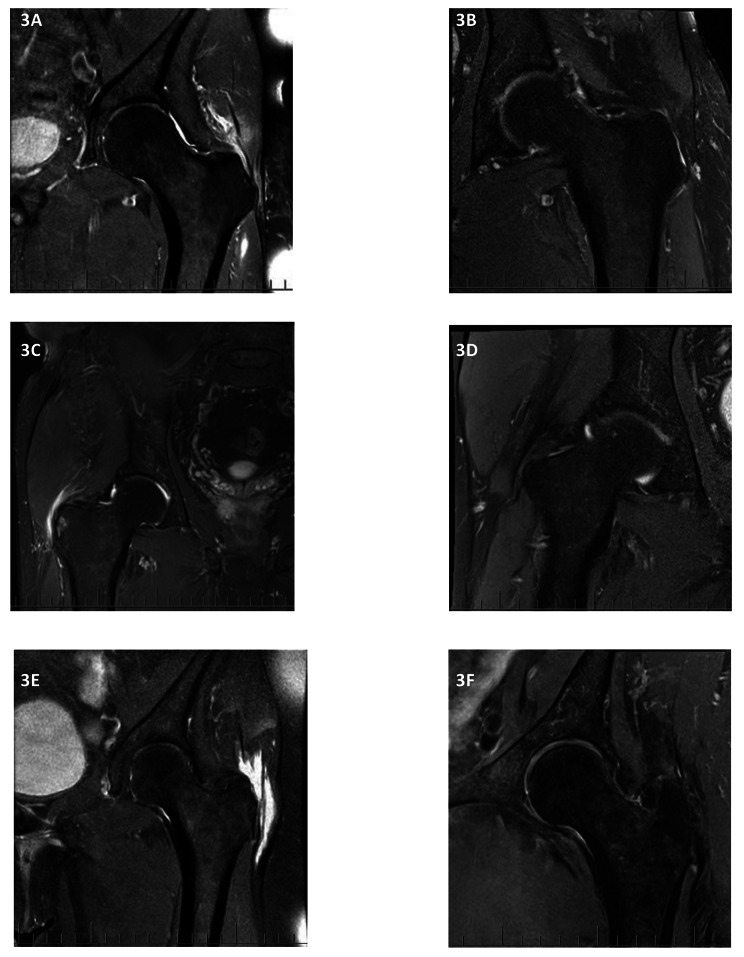
Pre- and post- MRI images from three participants who had improvement of more than 2 points in their MHIP scores. **3A** and **3B** are the pre- and post-treatment scans for participant 64; **3C** and **D** for participant 70, and **3E** and **3F** for participant 33 respectively

### Change in MHIP score elements

The MHIP score is calculated based on 5 elements although the total score reflects tendinopathy severity : gluteal tendinopathy rating (GT) and trochanteric bursitis (TB), cortical irregularity (CI) and bone marrow oedema (BO) and fatty muscular atrophy (FA) [12].

Tendon related scores were similar between the CSI group and LR-PRP groups, with the CSI group showing a small reduction in GT scores (1.7 (SD 0.6) to 1.3 (SD 0.6)) but no change in TB scores and the LR-PRP group showing no change in GT scores but a small reduction in TB scores (2.0 (SD 1.0) to 1.6 (SD 1.1)) (supplementary tables S1 and S2).

The bone related scores had the largest between group difference with CI increasing in the CSI group (0.3 (SD 0.6) to 0.6 (SD 0.6)) but decreasing in the LR-PRP group (0.6 (SD 0.7) to 0.4 (0.5)) and BO also reducing in the LR-PRP group (0.2 (SD 0.4) to 0 (SD 0)) (supplementary tables S1 and S2).

Fatty atrophic muscular changes were the same in the CSI group pre- and post- treatment (0.6 SD 1.2) and similar in the LR-PRP group (0.5 (SD 0.9) to 0.6 (SD 0.9)) (supplementary tables S1 and S2). Figure 3 shows the pre and post MRI images from three participants who had improvement of more than 2 points in their MHIP scores.

## Discussion

Our hypothesis was that there would be no improvement in MHIP MRI scores following treatment for gluteal tendinopathy at 2 years or more post-treatment. Overall, the results confirmed this. However, there was a reduction in the mean MHIP scores in the LR-PRP group from 5.3 (SD 3.0) to 4.77 (SD 2.5), p = 0.56) and in five out of nine participants the MHIP score improved. Four of these improved by 2–4 points whilst *no* participants in either of the CSI or CSI + LR-PRP groups experienced improvement of more than 2 points.

Our study is unique in that we analyzed MRI between at least two and up to seven years post-treatment (mean follow-up time; 4.15 ± 1.11 years). There are limited studies which look at structural change in tendinopathic tendons post biological injection treatment, and even fewer following patients in the longer-term. In their 2011 RCT, de Vos et al., showed that that there was no difference between the effect of PRP or placebo on structural changes and neovascularization in tendinopathic achilles tendons using ultrasound imaging [16]. This study followed participants (n = 52) out to 6-months post-treatment [16]. Other studies using ultrasound to measure structural change have shown minor changes in ultrasound characteristics at 6 and 12 months [17–20]. Bucher et al. looked at the effect of autologous tenocyte injection in 12 participants with gluteal tendinopathy [10]. This study demonstrated improvement in clinical outcome scores but MRI changes were not demonstrated in most cases at between 6 and 12 months post treatment [10]. Wang et al. is the only study providing long-term radiological follow-up of participants (n = 15) on autologous tenocyte implantation in tennis elbow [11, 21]. There was a statistically significant improvement in MRI scores post-treatment [21]. Notably, imaging was taken at a mean time of 4.51 (range 3.08–5.17) years post-treatment [21].

To date, tendon clinicians and researchers have a focused on measuring symptomatic rather than structural outcomes to determine improvement in tendinopathy. However, a recent consensus statement identified that almost 70% of tendinopathy researchers felt that structural analysis should be a core domain for tendinopathy assessment [22]. We postulate here that structural outcome measures are an important indicator of improvement in tendinopathy – and that the timepoint at which imaging is undertaken is crucial.

It is possible that effectiveness of treatment in tendinopathy is grade or stage dependent. A recent review of 27 studies gluteal tendinopathy by Ladurner et al., identified good [23]evidence for PRP in grade 1 and 2 tendinopathy. This aligns with the histopathological model by Bhabra et al. which suggested that PRP and autologous injections are likely to be effective treatments in early to moderate stage tendinopathy [6]. These treatments rely on stimulating tenocytes thus can be expected to be more effective before cell apoptosis and tenocyte depletion has occurred in Grade 3–4 tendinopathy. Future studies looking for structural improvement would benefit from ensuring stage appropriate treatments are included in the analysis.

Timing – both at imaging – and disease stage is likely to be important because time is required for tendons to undergo structural changes following treatment. This would corroborate patient-reported outcome measures from the original RCT by Fitzpatrick et al. which found that participants in the LR-PRP group continued to experience symptomatic improvement beyond 12 months and even further at final follow up at 24 months [8, 9]. It also aligns with evidence from carbon-dating studies which show that collagen turnover can take a long time – up to 15 years in tendinopathic tendons.

Our study was strengthened by use of a reliable grading score for gluteal tendinopathy as a method of comparing disease progression before and after receiving treatment. The MHIP score enables researchers to compare radiological findings pre- and post-treatment, between patient cohorts and across multiple studies [12].

Limitations of our study include that the study extension did not control for patients having subsequent interventions following completion of the trial. The sample size of the study was limited by the availability of baseline MRI scans from the RCT and thus is too small for ensuring significance and the results should be interpreted as hypothesis generating.

## Conclusion

The hypothesis that there would be no improvement in MHIP scores following treatment of gluteal tendinopathy was supported. Findings of improvement in the LR-PRP group at 4 years would support further studies powered to look for structural improvement. These findings suggest that structural change following treatment for tendinopathy may be possible supporting the inclusion of MRI as a core outcome for future studies.

### Electronic supplementary material

Below is the link to the electronic supplementary material.

Supplementary Material 1

## Data Availability

Data relating to this study has been published as a part of the article and the supplementary material.
