# Supplementary material for: Is there structural change on MRI in gluteal tendinopathy after treatment? Single outcome measure extension of an RCT
Source: BMC Med Imaging. 2023 Nov 8;23:179. doi: 10.1186/s12880-023-01150-y (PMC10634075; doi:10.1186/s12880-023-01150-y)
Supplement: Supplementary file 1 — Supplementary Material 1 [file 12880_2023_1150_MOESM1_ESM.docx]

# Title: Is there structural change on MRI in gluteal tendinopathy after treatment? Single outcome measure extension of an RCT.

Supplementary Tables

# Table S1. Classical 2-tailed, paired t-test comparing Mean ± SD MHIP scores for CSI-only group.

| Number of patients, 3 | Pre-treatment score | | Post-treatment score | p-value |
| --- | --- | --- | --- | --- |
| MHIP overall, mean ± SD | 4.333333 ± 2.309401 | 4.333333 ± 1.154701 | | 1.000 |
| MHIP elements, mean ± SD |  |  | |  |
| GT | 1.666667 ± .5773503 | 1.333333 ± .5773503 | | 0.6667 |
| FA | .6666667 ± 1.154701 | .6666667 ± 1.154701 | | 1.0000 |
| TB | 1.666667± .5773503 | 1.666667 ± .5773503 | | 1.0000 |
| CI | .3333333 ± .5773503 | .6666667 ± .5773503 | | 0.4226 |
| BO | 0 | 0 | |  |
| MHIP, Melbourne HIP MRI Score; GT, gluteal tendinopathy; TB, trochanteric bursitis; FA, fatty atrophy; CI, cortical irregularity; BO, bone marrow oedema; LR-PRP, leukocyte-rich platelet-rich plasma; CSI, corticosteroid injection. Note- a negative MHIP change indicates improvement and positive worsening. | | | | |

# Table S2. Classical 2-tailed, paired t-test comparing Mean ± SD MHIP scores for LR-PRP-only group.

| Number of patients, 9 | Pre-treatment score | Post-treatment score | p-value |
| --- | --- | --- | --- |
| MHIP overall, mean ± SD | 5.333333 ± 3.041381 | 4.777778 ± 2.488864 | 0.5604 |
| MHIP elements, mean ± SD |  |  |  |
| GT | 2.111111 ± 1.054093 | 2.111111 ± 1.166667 | 1.0000 |
| FA | .4444444 ± .8819171 | .5555556 ± .8819171 | 0.6811 |
| TB | 2 ± 1 | 1.666667 ± 1.118034 | 0.3466 |
| CI | .5555556 ± .7264832 | .4444444 ± .5270463 | 0.5943 |
| BO | .2222222 ± .4409586 | 0 | 0.1690 |
| MHIP, Melbourne HIP MRI Score; GT, gluteal tendinopathy; TB, trochanteric bursitis; FA, fatty atrophy; CI, cortical irregularity; BO, bone marrow oedema; LR-PRP, leukocyte-rich platelet-rich plasma; CSI, corticosteroid injection. Note- a negative MHIP change indicates improvement and positive worsening. | | | |

| **Table S3. MHIP scores in ascending order of difference in pre and post overall scores.** | | | | | | | | | | | | | |
| --- | --- | --- | --- | --- | --- | --- | --- | --- | --- | --- | --- | --- | --- |
|  | MHIP elements | | | | | | | | | | MHIP overall | |  |
| Patient ID | GT | | TB | | FA | | CI | | BO | |  | | Change |
|  | Pre | Post | Pre | Post | Pre | Post | Pre | Post | Pre | Post | Pre | Post |  |
| LR-PRP | | | | | | | | | | | | | |
| 64 | 3 | 1 | 3 | 1 | 2 | 2 | 1 | 1 | 0 | 0 | 9 | 5 | -4 |
| 70 | 3 | 1 | 2 | 1 | 0 | 0 | 0 | 0 | 1 | 0 | 6 | 2 | -4 |
| 33 | 3 | 3 | 4 | 4 | 2 | 1 | 2 | 1 | 0 | 0 | 11 | 9 | -2 |
| 74 | 2 | 1 | 2 | 1 | 0 | 0 | 0 | 0 | 0 | 0 | 4 | 2 | -2 |
| 4 | 2 | 2 | 1 | 1 | 0 | 0 | 1 | 1 | 1 | 0 | 5 | 4 | -1 |
| 25 | 2 | 3 | 2 | 1 | 0 | 2 | 1 | 0 | 0 | 0 | 5 | 6 | 1 |
| 68 | 0 | 1 | 1 | 1 | 0 | 0 | 0 | 0 | 0 | 0 | 1 | 2 | 1 |
| 76 | 3 | 4 | 1 | 2 | 0 | 0 | 0 | 0 | 0 | 0 | 4 | 6 | 2 |
| 58 | 1 | 3 | 2 | 3 | 0 | 0 | 0 | 1 | 0 | 0 | 3 | 7 | 4 |
| Mean ± SD | 2.1 ± 1.1 | 2.1 ± 1.1 | 2 ± 1 | 1.7 ± 1.1 | 0.4 ± 0.9 | 0.5 ± 0.9 | 0.6 ± 0.7 | 0.4 ± 0.5 | 0.2 ± 0.4 | 0 ± 0 | 5.3 ± 3.0 | 4.8 ± 2.5 |  |
| CSI | | | | | | | | | | | | | |
| 27 | 2 | 1 | 2 | 1 | 2 | 2 | 1 | 1 | 0 | 0 | 7 | 5 | -2 |
| 61 | 2 | 1 | 1 | 2 | 0 | 0 | 0 | 0 | 0 | 0 | 3 | 3 | 0 |
| 80 | 1 | 2 | 2 | 2 | 0 | 0 | 0 | 1 | 0 | 0 | 3 | 5 | 2 |
| Mean ± SD | 1.7 ± 0.6 | 1.3 ± 0.6 | 1.7 ± 0.6 | 1.7 ± 0.6 | 0.7 ± 1.2 | 0.7 ± 1.2 | 0.3 ± 0.6 | 0.7 ± 0.6 | 0 ± 0 | 0 ± 0 | 4.3 ± 2.3 | 4.3 ± 1.2 |  |
| CSI + LR-PRP | | | | | | | | | | | | | |
| 54 | 3 | 2 | 4 | 2 | 0 | 2 | 1 | 0 | 0 | 0 | 8 | 6 | -2 |
| 65 | 3 | 2 | 2 | 2 | 0 | 0 | 1 | 0 | 0 | 0 | 6 | 4 | -2 |
| 6 | 2 | 2 | 2 | 1 | 0 | 0 | 0 | 0 | 0 | 0 | 4 | 3 | -1 |
| 20 | 1 | 1 | 1 | 1 | 0 | 0 | 0 | 1 | 0 | 0 | 2 | 3 | 1 |
| 1 | 0 | 2 | 1 | 1 | 0 | 0 | 0 | 0 | 0 | 0 | 1 | 3 | 2 |
| 71 | 2 | 4 | 2 | 2 | 0 | 0 | 0 | 1 | 0 | 0 | 4 | 7 | 3 |
| 78 | 1 | 3 | 1 | 2 | 0 | 0 | 1 | 1 | 0 | 0 | 3 | 6 | 3 |
| 69 | 1 | 3 | 1 | 2 | 0 | 1 | 0 | 0 | 0 | 0 | 2 | 6 | 4 |
| Mean ± SD | 1.6 ± 1.1 | 4.8 ± 1.7 | 1.8 ± 1.0 | 1.6 ± 5.2 | 0 ± 0 | 0.4 ± 0.7 | 0.4 ± 0.5 | 0.4 ± 0.5 | 0 ± 0 | 0 ± 0 | 3.8 ± 2.3 | 4.8 ± 1.7 |  |
| MHIP, Melbourne HIP MRI Score; GT, gluteal tendinopathy; TB, trochanteric bursitis; FA, fatty atrophy; CI, cortical irregularity; BO, bone marrow oedema; LR-PRP, leukocyte-rich platelet-rich plasma; CSI, corticosteroid injection. Pre, before treatment; Post, after treatment. Note- a negative MHIP change indicates improvement and positive worsening. | | | | | | | | | | | | | |
